# Evaluating Targeted Dementia Screening of High‐Risk Patients in Primary Care: Preliminary Results from the electronic health record Risk of Alzheimer's and Dementia Assessment Rule (eRADAR) pragmatic randomized trial

**DOI:** 10.1002/alz70858_099443

**Published:** 2025-12-25

**Authors:** Deborah E Barnes, Leah Karliner, Abisola Idu, Deborah King, Judith Walsh, Sei J Lee, Tyler D Barrett, Mikael Anne Greenwood‐Hickman, Clarissa W Hsu, R Yates Coley, Alana Elop, Leonardo Colemon, Sascha Dublin

**Affiliations:** ^1^ University of California, San Francisco, San Francisco, CA, USA; ^2^ Kaiser Permanente Washington Health Research Institute, Seattle, WA, USA; ^3^ San Francisco VA Health Care System, San Francisco, CA, USA

## Abstract

**Background:**

Approximately half of people with dementia and 90% with mild cognitive impairment (MCI) are undiagnosed, making it harder to access appropriate care. eRADAR is a validated algorithm that uses structured electronic health record (EHR) data to identify patients at risk of undiagnosed dementia (C statistics: 0.79 to 0.84). We performed embedded, pragmatic clinical trials at Kaiser Permanente Washington (KPWA) and the University of California, San Francisco (UCSF) to assess the impact of targeted dementia screening using eRADAR on dementia detection and patient outcomes.

**Method:**

Study participants were aged 65+ without documented dementia (diagnoses or current dementia‐related medication) who had eRADAR scores in the top 15% (20% in Black/African American patients at UCSF to ensure equivalent sensitivity). They were randomized at the primary care provider (PCP) level to usual care or intervention. The intervention consisted of a “brain health” assessment visit with a clinical research interventionist embedded in the primary care clinic. Participants were encouraged to include a care partner. Visits could be in‐person or by video or phone. At UCSF, visits were offered in Spanish, Mandarin, and Cantonese in addition to English. The interventionist assessed functional status (independence in instrumental activities of daily living), depressive symptoms (PHQ‐2/9), and cognitive function (Montreal Cognitive Assessment) and shared the findings with the participant and their PCP. The PCP independently followed up and made diagnoses at their discretion.

**Result:**

3417 KPWA and 1271 UCSF patients were identified as “high risk” based on their eRADAR scores and randomized to targeted outreach or usual care. Of those who received outreach, 29% at KPWA and 31% at UCSF completed an assessment visit. Visit completion rates were 52% to 74% lower in those who were older vs. younger and Black or Asian (both sites) or Spanish‐speaking (UCSF only) vs. non‐Hispanic White. Of those who completed the visit, approximately half had results consistent with undiagnosed cognitive impairment (KPWA: 17% possible dementia, 32% MCI; UCSF: 21% possible dementia, 31% MCI).

**Conclusion:**

Among older adults identified as high‐risk using eRADAR who agreed to an assessment visit, about half appeared to have undiagnosed cognitive impairment. Additional studies are needed to explore disparities in visit acceptance.

